# Development of a core outcome set for myelodysplastic syndromes – a Delphi study from the EUMDS Registry Group

**DOI:** 10.1111/bjh.16654

**Published:** 2020-05-14

**Authors:** Ursula Rochau, Igor Stojkov, Annette Conrads‐Frank, Helena H. Borba, Karin A. Koinig, Marjan Arvandi, Corine van Marrewijk, Hege Garelius, Ulrich Germing, Argiris Symeonidis, Guillermo F. Sanz, Pierre Fenaux, Theo de Witte, Fabio Efficace, Uwe Siebert, Reinhard Stauder

**Affiliations:** ^1^ Department of Public Health, Health Services Research and Health Technology Assessment Institute of Public Health, Medical Decision Making and Health Technology Assessment UMIT – University for Health Sciences, Medical Informatics and Technology Hall i.T. Austria; ^2^ Department of Pharmacy Pharmaceutical Sciences Postgraduate Research Program Federal University of Paraná Curitiba Brazil; ^3^ Department of Internal Medicine V (Hematology and Oncology) Innsbruck Medical University Innsbruck Austria; ^4^ Department of Hematology Radboud university medical center Nijmegen the Netherlands; ^5^ Department of Medicine Section of Hematology and Coagulation Sahlgrenska University Hospital Göteborg Sweden; ^6^ Department of Hematology, Oncology and Clinical Immunology Universitätsklinik Düsseldorf Düsseldorf Germany; ^7^ Department of Internal Medicine Division of Hematology University of Patras Medical School Patras Greece; ^8^ Department of Hematology Hospital Universitario y Politécnico La Fe Valencia Spain; ^9^ Centro de Investigación Biomédica en Red de Cáncer CIBERONC Instituto de Salud Carlos III Madrid Spain; ^10^ Service d'Hématologie Hôpital Saint‐Louis Assistance Publique des Hôpitaux de Paris (AP‐HP) and Université Paris 7 Paris France; ^11^ Department of Tumor Immunology ‐ Nijmegen Center for Molecular Life Sciences Radboud university medical center Nijmegen the Netherlands; ^12^ Health Outcomes Research Unit Gruppo Italiano Malattie Ematologiche dell’Adulto (GIMEMA) Rome Italy; ^13^ Center for Health Decision Science Department of Health Policy and Management Harvard T.H. Chan School of Public Health Boston MA USA; ^14^ Institute for Technology Assessment and Department of Radiology Massachusetts General Hospital Harvard Medical School Boston MA USA

**Keywords:** myelodysplastic syndromes (MDS), core outcome set (COS), Delphi survey, outcome study, clinical trial

## Abstract

Treatment options for myelodysplastic syndromes (MDS) vary widely, depending on the natural disease course and patient‐related factors. Comparison of treatment effectiveness is challenging as different endpoints have been included in clinical trials and outcome reporting. Our goal was to develop the first MDS core outcome set (MDS‐COS) defining a minimum set of outcomes that should be reported in future clinical studies. We performed a comprehensive systematic literature review among MDS studies to extract patient‐ and/or clinically relevant outcomes. Clinical experts from the European LeukemiaNet MDS (EUMDS) identified 26 potential MDS core outcomes and participated in a three‐round Delphi survey. After the first survey (56 experts), 15 outcomes met the inclusion criteria and one additional outcome was included. The second round (38 experts) resulted in six included outcomes. In the third round, a final check on plausibility and practicality of the six included outcomes and their definitions was performed. The final MDS‐COS includes: health‐related quality of life, treatment‐related mortality, overall survival, performance status, safety, and haematological improvement. This newly developed MDS‐COS represents the first minimum set of outcomes aiming to enhance comparability across future MDS studies and facilitate a better understanding of treatment effectiveness.

Myelodysplastic syndromes (MDS) are characterised by ineffective haematopoiesis, abnormal cell morphology and increased risk of leukaemic evolution.^1^ Several treatment options are available, including supportive therapy, growth factors, disease‐modifying modalities, such as intensive antileukaemic chemotherapy and allogeneic haematopoietic stem cell transplantation (HSCT).^1^, ^2^, ^3^ Standardised reporting of outcomes is therefore essential. In 2000, the International Working Group (IWG) developed standardised response criteria^4^ for MDS, revised in 2006,^5^ as an important step toward standardising outcome measurements. The IWG response criteria mainly focus on haematological improvement and criteria for altering the natural history of disease, such as remission, treatment failure, progression and survival.^4^, ^5^ Since alleviation of symptoms represents a relevant goal in the treatment of patients with MDS, the inclusion of scores to assess health‐related quality of life (HRQoL) has been suggested in the original version of IWG.^4^ However, the definition of endpoints in MDS is complex, resulting in heterogeneity of outcomes used across different studies.

Reporting bias has frequently been observed in haematological studies and in clinical trials performed on MDS patients,^6^, ^7^ and comparison of treatment effectiveness is therefore challenging.^8^, ^9^ For the evaluation of evidence‐based treatment effectiveness, the validity of evidence syntheses is an essential factor. The validity is severely limited by non‐standardised outcome reporting. Selective reporting of outcomes within studies can be so diverse that it may have major negative implications on treatment recommendations.^10^ Additionally, quantitative evidence synthesis, such as meta‐analysis, indirect treatment comparison, or cost‐effectiveness analysis are required in most health technology assessments of recently introduced treatments.^11^ Comparison of such assessments may become impossible if different outcome measures are used across studies. Standardised reporting increases the comparability and transparency of research, decreasing unnecessary or overlapping research, which may be regarded as unethical.^12^, ^13^

A systematic approach to developing a common understanding of crucial outcome criteria is a core outcome set (COS) which includes a minimum set of relevant outcomes (i.e., study endpoints). The Core Outcome Measures in Effectiveness Trials (COMET) initiative^14^ published general guidelines for developing and reporting a COS. A COS represents an ‘agreed minimum set of outcomes that should be measured and reported in all clinical trials of a specific disease or trial population’.^14^, ^15^ It could provide better comparability between the outcome measurements across studies, strengthening the evidence pool and increasing the overall validity of therapy evaluation, leading to more reliable treatment recommendations.^1^ A recent review showed that more than 200 studies across various diseases are published on the application or development of methodology to determine how relevant outcomes should be selected, defined and measured.^16^

In addition to uniform reporting, an increasingly important aspect of cancer patients’ treatment is HRQoL.^17^ The value of assessing Patient‐Reported Outcomes (PROs) in MDS has been emphasised in recent international guidelines,^1^ as empirical evidence has clearly demonstrated major HRQoL impairments and a substantial symptom burden.^18^, ^19^, ^20^ Notably, PROs are now included amongst the four types of clinical outcome assessments by the US Food and Drug Administration (FDA) which can be used to determine treatment benefit of a new drug.^21^ Therefore, integrating HRQoL and other types of PROs into a standardised MDS‐COS is highly relevant for patient‐centered care.

The aim of our study was to establish a consensus‐based MDS‐COS, including traditional clinical outcomes and PROs. This study focuses on the identification and standardisation of the minimum outcome measurements for clinical studies from the clinical perspective of MDS experts.

## Material and methods

Our project is part of the of MDS‐RIGHT Work Package 3 ‘Health‐related quality of life issues in elderly patients with anaemia’. MDS‐RIGHT (https://mds‐europe.eu/right) is a European Horizon 2020 project which evolved from the European LeukemiaNet and has been launched by the European LeukaemiaNet MDS (EUMDS) registry.^22^

Our project was structured in three phases, including outcome identification, Delphi survey rounds, and definition of the COS.

### Phase 1: Outcome identification

**Fig 1:**
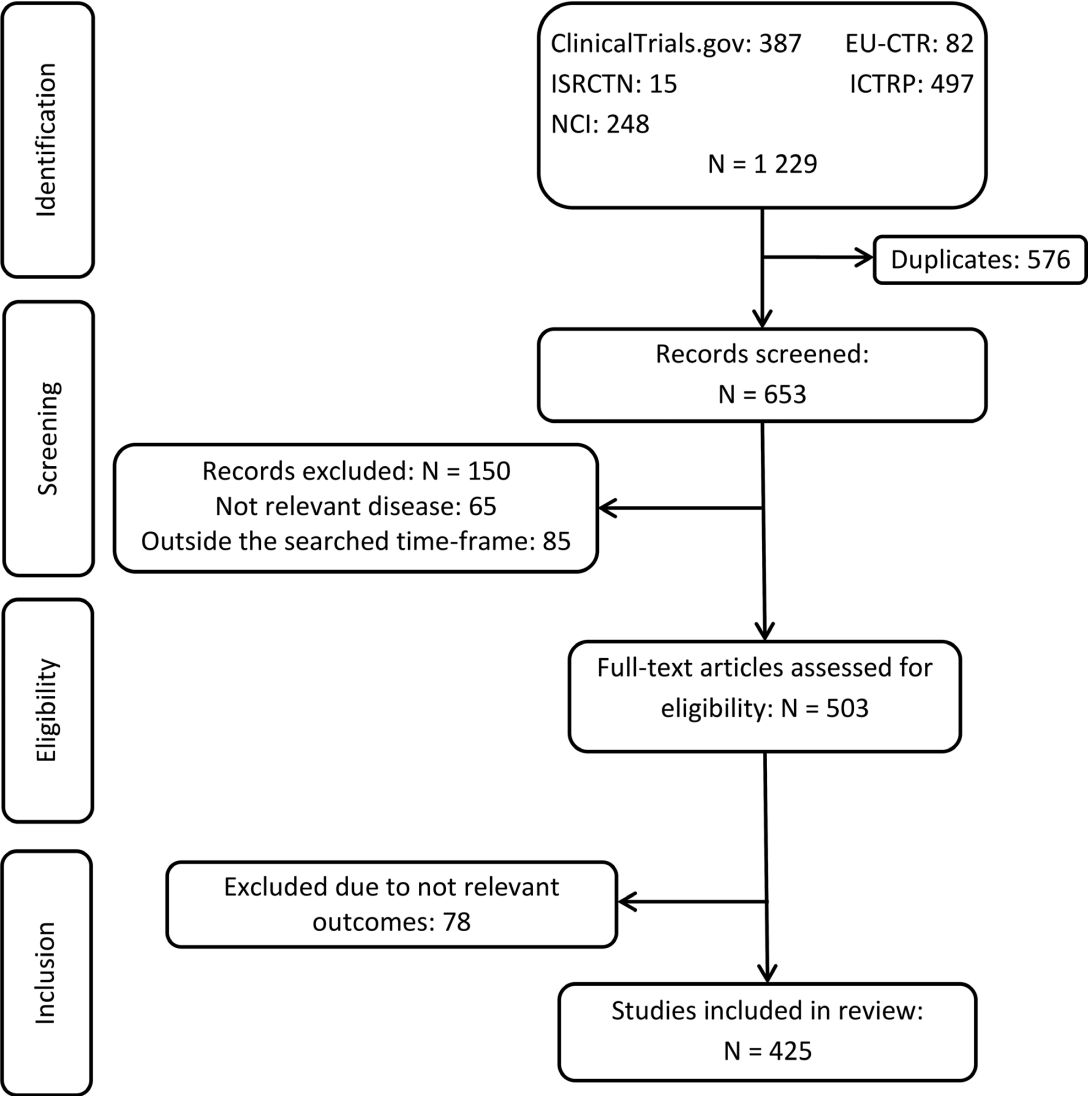
Flow chart representing study identification and selection process. Summary of the systematic literature review to identify potential MDS outcomes to be included in the core outcome set. The systematic literature review included the following four phases: identification, screening, eligibility and inclusion. EU‐CTR, European Union Clinical Trials Register; ICTRP: International Clinical Trials Registry Platform; ISRCTN, International Standard Registered Clinical/soCial sTudy Number registry; N, number of studies; NCI, National Cancer Institute Registry.

We conducted a systematic review of observational and interventional studies in MDS patients to provide an overview of all potential outcomes for a MDS‐COS. The systematic literature review was performed in the ClinicalTrials.gov database and four clinical trial registries (International Clinical Trials Registry Platform, National Cancer Institute Clinical Trials Database, World Health Organisation International Clinical Trials Registry Platform and The European Union Clinical Trials Register). Studies published in English, registered up to four years prior to the survey (January 2012–January 2016), including MDS patients, regardless of the number of patients enrolled or the classification system used, were included. Studies focusing solely on pharmacodynamics, pharmacokinetics, or molecular research were not considered eligible for inclusion (Fig 1).

Two of our researchers (Igor Stojkov, Helena H. Borba) screened studies and performed data extractions. When questions arose on study eligibility, further researchers were consulted (Ursula Rochau, Reinhard Stauder). Data on the registration year, intervention, study population characteristics, and primary and secondary outcomes were extracted and summarised in a comprehensive evidence table. After excluding duplicates, the outcomes reported in the included studies were categorised into similar domains to develop and label potential core outcomes. Together with clinical MDS experts, researchers actively participated in the discussion and came to a consensus on the potential MDS core outcomes.

### Phase 2: Questionnaire development and Delphi survey rounds

The potential outcomes identified in phase 1 formed the basis for phase 2, i.e., an anonymous online questionnaire using Google Forms, which was used in the Delphi survey.^23^, ^24^ Experts participating in the Delphi survey were recruited from the MDS‐RIGHT project and the EUMDS (European MDS) Registry, including clinicians, operational team members, data managers, statisticians, health science researchers and research nurses.

The questionnaire contained four sections: project description and participant demographics, ranking scale for the importance of including each outcome in the MDS‐COS, additional outcomes and comments, and the consent form. Additionally, for every outcome an explanation was given by a general, non‐MDS‐specific definition. As suggested by the Grading of Recommendations Assessment, Development and Evaluations (GRADE) scale,^25^ we used a nine‐point Likert scale (1–3: low importance for decision‐making, 4–6: important but not critical for decision‐making; 7–9: critical for decision‐making) to rank importance. In this round, we also asked the participating experts for each outcome to select the application area most relevant for that outcome. The following three application areas were included and each application area had three subcategories: patients’ MDS risk group by the Revised International Prognostic Scoring System (IPSS‐R) (subcategories: 0–3; >3–4·5; >4·5),^26^ treatment (subcategories: supportive/disease modifying/HSCT), and clinical setting (subcategories: clinical study/registry/daily practice). This selection was optional and participants were allowed to choose more than one subcategory. A likelihood ratio χ^2^ test was performed to analyse differences in the relevance of a specific outcome within an application area. For example, it was evaluated whether a specific outcome is more relevant for IPSS‐R low‐risk patients compared to high‐risk patients. The likelihood ratio χ^2^ test compared the likelihood ratios of the random‐effect logit intercept model with the polytomous variable random‐effect logit model. Differences in the relevance between the subcategories within a specific application were considered statistically significant if the *P* < 0·05. Additional outcomes suggested by at least two experts were included in the second Delphi round. Survey questionnaires are presented in the supplement.

In the second Delphi round, newly suggested outcomes and the results from the previous ranking were added to the questionnaire. These results provided the opportunity for experts to change their opinion in terms of the group’s ranking. Based on the COMET initiative’s recommendation, outcomes ranked 7–9 by at least 70% of the experts, and not ranked 1–3 by more than 15%, were included.^27^ Additionally, outcomes were excluded if ranked 1–3 by at least 70% of the experts, and not ranked 7–9 by more than 15%. All remaining outcomes not fulfilling the inclusion or exclusion criteria were labelled ‘without consensus’.

### Phase 3: Confirmation and outcome‐defining round

The final focus round of the Delphi survey aimed at deriving a final confirmation, checking for plausibility and practicality, and defining the included outcomes. An online open document was created, listing the details from the previous rankings, as well as the remaining open questions and general instructions. As planned, a smaller group of experienced MDS experts was invited to enable active discussions. The open access of the document enabled the experts to actively follow the ongoing discussion and to conclude the development of the MDS‐COS.

### Study management and ethics

The survey distribution was facilitated by an independent researcher (Corine van Marrewijk).

The study received ethical approval from the Research Committee for Scientific and Ethical Questions at the UMIT ‐ University for Health Sciences, Medical Informatics and Technology, Hall in Tirol, Austria. EUMDS (ClinicalTrials.gov: NCT00600860) has been approved by the ethics committees of all participating centres and is performed in accordance with the Declaration of Helsinki.

## Results

### Phase 1: Outcome identification

In the systematic review, 1229 studies were identified. After removal of duplicates, 653 studies entered the screening process, of which 425 studies fulfilled the inclusion criteria (Fig 1). Included studies were mainly interventional studies which evaluated drug efficacy and effectiveness (Table S1). The extracted data were summarised in 1341 primary and secondary outcomes, which were condensed due to the broad overlap, and operationalised into the following 26 MDS core outcome candidates: overall survival (OS), HRQoL, duration of hospitalisation, cytogenetic response, haematological improvement, response/remission, time to response, overall response, safety, adverse event, infectious event, iron overload, secondary morbidity, need for supportive therapy, treatment‐related mortality, acute/chronic graft‐versus‐host disease, event‐free survival, failure‐free survival, disease‐free survival, relapse‐free survival, progression‐free survival, time to progression, performance status, functional activities, number of transfusions per patient, and need for HSCT.

### Phase 2: Survey results

Fifty‐six MDS experts from 14 different countries participated at the first Delphi round.

After the first round of ranking, the following 15 outcomes were considered to be highly important (7–9) by more than 70% of the experts: treatment‐related mortality, haematological improvement, OS, response/remission, performance status, safety, HRQoL, need for HSCT, acute/chronic graft‐versus‐host disease, progression‐free survival, overall response, number of transfusions per patient, adverse event, need for supportive therapy, and time to progression. One new outcome (secondary malignancy) was suggested by two experts. None of the outcomes was excluded (ranked 1–3 by more than 70% of the experts). In addition to the ranking of the importance of the outcomes, experts were asked to assess the application area in which the outcome is most relevant {i.e., for patients with a specific IPSS‐R risk group [subcategories: (Very) Low 0–3; Intermediate >3–4·5; (Very) High >4·5], a specific therapy [subcategories: supportive, disease‐modifying, HSCT], and specific clinical setting [subcategories: clinical study, registry, and daily practice]}. For the majority of outcomes (81%), we found statistically significant differences in relevance within a specific application area. For example, many of the outcomes were considered more relevant when patients had been treated with disease‐modifying drugs or HSCT, compared to supportive care. Results for the assessment of relevance are summarised in Table 1.

**Table 1:** Application of outcomes: results from the first Delphi round.

Potential MDS core outcomes	IPSS‐R risk group				Therapy				Clinical setting			
	0–3 (Very) Low	>3–4·5 Intermediate	>4·5 (Very) High	*, ‡	Supportive	Disease‐modifying	HSCT	*, ‡	Clinical study	Registry	Daily practice	*, ‡
**Health‐related quality of life**	41 (34·5)	38 (31·9)	40 (33·6)		37 (35·9)	37 (35·9)	29 (28·2)		30 (33·7)	21 (23·6)	38 (42·7)	*, ‡
**Treatment‐related mortality**	25 (23·4)	34 (31·8)	48 (44·9)	*, ‡	11 (11·8)	37 (39·8)	45 (48·4)	*, ‡	33 (41·8)	20 (25·3)	26 (32·9)	*, ‡
**Overall survival**	28 (25·5)	35 (31·8)	47 (42·7)	*, ‡	14 (14·3)	41 (41·8)	43 (43·9)	*, ‡	34 (38·6)	27 (30·7)	27 (30·7)	
**Performance status**	38 (29·5)	45 (34·9)	46 (35·7)	*, ‡	23 (20·9)	41 (37·3)	46 (41·8)	*, ‡	38 (39·2)	24 (24·7)	35 (36·1)	*, ‡
**Safety**	46 (35·9)	42 (32·8)	40 (31·3)		24 (24·0)	41 (41·0)	35 (35·0)	*, ‡	38 (43·2)	18 (20·5)	32 (36·4)	*, ‡
**Haematological improvement**	40 (32·8)	43 (35·2)	39 (32·0)		21 (26·3)	40 (50·0)	19 (23·8)	*, ‡	31 (35·6)	22 (25·3)	34 (39·1)	*, ‡
Adverse event	40 (32·8)	42 (34·4)	40 (32·8)		23 (22·5)	42 (41·2)	37 (36·3)	*, ‡	38 (45·2)	18 (21·4)	28 (33·3)	*, ‡
Functional activities	41 (36·0)	36 (31·6)	37 (32·5)		26 (28·0)	33 (35·5)	34 (36·6)		31 (41·3)	16 (21·3)	28 (37·3)	*, ‡
Response/Remission	17 (17·2)	32 (32·3)	50 (50·5)	*, ‡	8 (9·3)	39 (45·3)	39 (45·3)	*, ‡	34 (45·9)	20 (27·0)	20 (27·0)	*, ‡
Progression‐free survival	29 (25·7)	38 (33·6)	46 (40·7)	*, ‡	18 (18·2)	43 (43·4)	38 (38·4)	*, ‡	33 (41·8)	21 (26·6)	25 (31·6)	*, ‡
Time to progression	35 (29·2)	42 (35·0)	43 (35·8)		16 (17·8)	41 (45·6)	33 (36·7)	*, ‡	35 (43·8)	22 (27·5)	23 (28·8)	*, ‡
Need for supportive therapy	42 (38·2)	38 (34·5)	30 (27·3)	*, ‡	28 (34·6)	28 (34·6)	25 (30·9)		28 (39·4)	16 (22·5)	27 (38·0)	*, ‡
Overall response	31 (25·8)	41 (34·2)	48 (40·0)	*, ‡	13 (15·3)	41 (48·2)	31 (36·5)	*, ‡	33 (42·9)	20 (26·0)	24 (31·2)	*, ‡
Acute/Chronic GvHD	10 (15·9)	22 (34·9)	31 (49·2)	*, ‡	3 (5·0)	6 (10·0)	51 (85·0)	*, ‡	20 (40·8)	15 (30·6)	14 (28·6)	
Need for HSCT	8 (9·4)	28 (32·9)	49 (57·6)	*, ‡	3 (6·3)	17 (35·4)	28 (58·3)	*, ‡	27 (41·5)	22 (33·8)	16 (24·6)	*, ‡
No. of transfusions per patient	47 (40·9)	43 (37·4)	25 (21·7)	*, ‡	37 (45·1)	26 (31·7)	19 (23·2)	*, ‡	28 (35·9)	22 (28·2)	28 (35·9)	
Infectious event	30 (27·0)	37 (33·3)	44 (39·6)	*, ‡	24 (22·9)	40 (38·1)	41 (39·0)	*, ‡	35 (40·2)	18 (20·7)	34 (39·1)	*, ‡
Relapse‐free survival	16 (17·8)	30 (33·3)	44 (48·9)	*, ‡	10 (11·5)	36 (41·4)	41 (47·1)	*, ‡	33 (50·0)	15 (22·7)	18 (27·3)	*, ‡
Disease‐free survival	21 (22·3)	29 (30·9)	44 (46·8)	*, ‡	12 (13·6)	38 (43·2)	38 (43·2)	*, ‡	35 (51·5)	16 (23·5)	17 (25·0)	*, ‡
Secondary morbidity	40 (40·4)	35 (35·4)	24 (24·2)	*, ‡	23 (26·1)	31 (35·2)	34 (38·6)	*, ‡	33 (37·9)	23 (26·4)	31 (35·6)	
Duration of hospitalisation	24 (25·8)	30 (32·3)	39 (41·9)	*, ‡	23 (24·7)	35 (37·6)	35 (37·6)	*, ‡	27 (40·9)	16 (24·2)	23 (34·8)	
Failure‐free survival	22 (23·7)	30 (32·3)	41 (44·1)	*, ‡	13 (14·9)	38 (43·7)	36 (41·4)	*, ‡	33 (47·8)	19 (27·5)	17 (24·6)	*, ‡
Event‐free survival	27 (24·8)	35 (32·1)	47 (43·1)	*, ‡	16 (17·2)	39 (41·9)	38 (40·9)	*, ‡	36 (49·3)	20 (27·4)	17 (23·3)	*, ‡
Time to response	26 (24·8)	34 (32·4)	45 (42·9)	*, ‡	7 (11·1)	36 (57·1)	20 (31·7)	*, ‡	30 (50·0)	13 (21·7)	17 (28·3)	*, ‡
Iron overload	47 (47·0)	38 (38·0)	15 (15·0)	*, ‡	32 (45·7)	12 (17·1)	26 (37·1)	*, ‡	22 (32·4)	23 (33·8)	23 (33·8)	
Cytogenetic response	16 (17·8)	28 (31·1)	46 (51·1)	*, ‡	2 (2·6)	37 (47·4)	39 (50·0)	*, ‡	33 (49·3)	17 (25·4)	17 (25·4)	*, ‡

The six outcomes which were finally included in the COS are in bold. First round participants (*n* = 56) were asked for which patients' MDS risk groups, which therapy and in which clinical setting (i.e., clinical study, registry or daily practice) the outcomes are most relevant. The table shows how many participants recommended the application of a specific outcome for a specific situation. The numbers in parentheses show the percentage of answers within one area (e.g., 34·5% out of all answers in the IPSS‐R category were in risk group 0–3 for the outcome HRQoL). The selection was optional and participants could choose more than one subcategory. Columns with * indicate a significant difference in the recommendation for one of the three subcategories within each area for a specific outcome, according to the results from the LR χ^2^‐Likelihood Ratio χ^2^ test.

GvHD, graft‐versus‐host disease; HSCT, haematopoietic stem cell transplantation; IPSS‐R, Revised International Prognostic Scoring System; MDS, myelodysplastic syndromes; HRQoL, health‐related quality of life; No., number.

* Statistically significant difference (*P* < 0·05).

In the second Delphi round, 38 experts completed questionnaires. From these responses, 19 experts had also taken part in the previous round. Mainly haematologists with longstanding clinical expertise in MDS participated in the surveys (Table 2).

**Table 2:** Characteristics of the MDS experts engaged in the Delphi survey.

	1^st^ Round *N* = 56	2^nd^ Round *N* = 38	3^rd^ Round *N* = 4
Male, *N* (%)	28 (50)	20 (52·6)	3 (75)
Age, mean (SD)	50·2 ± 9·6	52·4 ± 8	–
Country, *N* (%)			
Austria	2 (3·6)	3 (7·9)	1 (25)
Croatia	2 (3·6)	1 (2·6)	–
Czech Republic	3 (5·4)	3 (7·9)	–
Denmark	–	1 (2·6)	–
France	5 (8·9)	6 (15·8)	–
Germany	1 (1·8)	1 (2·6)	1 (25)
Greece	17 (30·4)	4 (10·5)	–
Israel	3 (5·4)	4 (10·5)	–
Italy	–	1 (2·6)	–
the Netherlands	–	1 (2·6)	1 (25)
Poland	2 (3·6)	–	–
Portugal	1 (1·8)	1 (2·6)	–
Romania	2 (3·6)	1 (2·6)	–
Serbia	1 (1·8)	2 (5·3)	–
Spain	10 (17·9)	2 (5·3)	–
Sweden	3 (5·4)	2 (5·3)	1 (25)
United Kingdom	4 (7·1)	5 (13·2)	–
Specialty, *N* (%)			
Haematology	50 (89·3)	30 (78·9)	1 (25)
Haematology & oncology	3 (5·4)	6 (15·8)	3 (75)
Internal medicine	3 (5·4)	1 (2·6)	–
Health outcomes research expert	–	1 (2·6)	–
Work experience, *N* (%)			
< 5 years	3 (5·4)	–	–
5–10 years	6 (10·7)	2 (5·3)	–
> 10 years	47 (83·9)	36 (94·7)	4 (100)
Experience with MDS patients, *N* (%)			
< 5 years	3 (5·4)	–	–
5–10 years	12 (21·4)	8 (21·1)	–
> 10 years	41 (73·2)	30 (78·9)	4 (100)

MDS, myelodysplastic syndromes; *N*, number of participants; SD, standard deviation.

In the second round, the following six outcomes met the inclusion criteria: HRQoL, treatment‐related mortality, OS, performance status, safety, and haematological improvement. No consensus was achieved on the remaining 21 outcomes.

**Fig 2:**
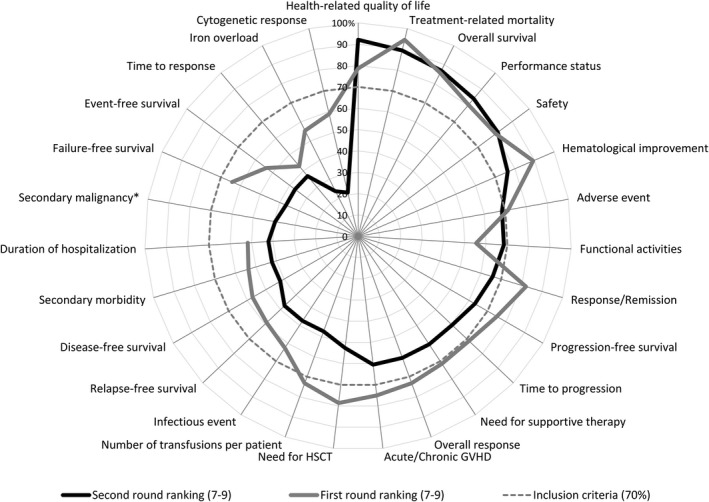
Outcome rankings (7–9) of the two Delphi rounds. The figure shows how often (in percentage) the ranking ‘highly important’ (7–9) was selected for each outcome by the survey participants of Delphi round one and two. *New outcome suggested after the first round. GvHD, graft‐versus‐host disease; HSCT, haematopoietic stem cell transplantation.

An overview of the two‐round outcome rankings 7–9 is illustrated in Fig 2, with more detailed information presented in Table 3.

**Table 3:** Results of the first and second Delphi survey rounds: rankings of the potential MDS core outcomes.

	1^st^ Round *N* = 56			2^nd^ Round *N* = 38		
Potential MDS core outcomes	Median (Range)	Ranking 1–3 in %	Ranking 7–9 in %	Median (Range)	Ranking 1–3 in %	Ranking 7–9 in %
Health‐related quality of life	8 (5–9)	0·0	78·6†	8 (4–9)	0·0	92·1*, ‡
Treatment‐related mortality	8 (5–9)	0·0	94·6†	8 (2–9)	2·6	89·5*, ‡
Overall survival	9 (5–9)	0·0	85·7†	8 (5–9)	0·0	86·8*, ‡
Performance status	7·5 (3–9)	1·8	80·4†	7·5 (3–9)	2·6	84·2*, ‡
Safety	8 (4–9)	0·0	80·4†	8 (4–9)	0·0	81·6*, ‡
Haematological improvement	8 (4–9)	0·0	89·3†	8 (3–9)	2·6	76·3*, ‡
Adverse event	8 (4–9)	0·0	71·4†	7 (1–9)	2·6	68·4
Functional activities	7 (1–9)	5·4	55·4	7 (2–9)	2·6	68·4
Response/remission	8 (4–9)	0·0	82·1†	7 (4–9)	0·0	65·8
Progression‐free survival	8 (3–9)	1·8	75·0†	7 (4–9)	0·0	63·2
Time to progression	8 (1–9)	3·6	71·4†	7 (4–9)	0·0	60·5
Need for supportive therapy	7 (3–9)	7·1	71·4†	7 (3–9)	2·6	60·5
Overall response	7 (1–9)	1·8	73·2†	7 (3–9)	7·9	60·5
Acute/chronic GvHD	8 (4–9)	0·0	75·0†	7 (1–9)	21·1	60·5
Need for HSCT	8 (1–9)	3·6	78·6†	7 (1–9)	18·4	52·6
Number of transfusions per patient	7 (3–9)	1·8	73·2†	6 (3–9)	2·6	47·4
Infectious event	7 (3–9)	1·8	62·5	6 (2–9)	2·6	47·4
Relapse‐free survival	7 (1–9)	3·6	58·9	6 (2–9)	5·3	47·4
Disease‐free survival	7 (3–9)	5·4	57·1	6 (1–9)	7·9	42·1
Secondary morbidity	7 (4–9)	0·0	53·6	6 (3–9)	7·9	42·1
Duration of hospitalisation	7 (3–9)	3·6	51·8	6 (1–9)	10·5	42·1
Secondary malignancy*	–	–	–	6 (1–9)	15·8	39·5
Failure‐free survival	7 (1–9)	5·4	64·3	6 (3–9)	2·6	36·8
Event‐free survival	7 (2–9)	7·1	53·6	6 (2–9)	10·5	36·8
Time to response	6 (2–9)	12·5	42·9	6 (3–9)	10·5	36·8
Iron overload	7 (1–9)	5·4	55·4	6 (1–9)	18·4	23·7
Cytogenetic response	7 (1–9)	7·1	58·9	5 (3–9)	18·4	21·1

GvHD, graft‐versus‐host disease; HSCT, haematopoietic stem cell transplantation; MDS, myelodysplastic syndromes; *N*, number of participants.

* New outcome suggested after the first round.

† Outcomes fulfilling the inclusion criteria after the first round of the Delphi survey.

‡ Outcomes fulfilling the inclusion criteria after the second round of the Delphi survey.

### Phase 3: Confirmation and outcome‐defining round

After the first two rounds which included a large number of experts, a selected focus group of four international MDS experts (HG, UG, RS, and TdW) participated in the final focus Delphi round. In this round, none of the 21 outcomes without consensus was proposed for re‐ranking and they were thereby finally excluded. The six outcomes (HRQoL, treatment‐related mortality, OS, performance status, safety and haematological improvement), which had been labelled as included after the second round, were retained and defined in relation to MDS. Table 4 presents the final COS, including detailed definitions for the application in MDS.

**Table 4:** Definitions of the MDS core outcomes.

Health‐related quality of life	General definition: Quality of life is described ‘as an individual's perception of their position in life in the context of the culture and value systems in which they live and in relation to their goals, expectations, standards and concerns. It is a broad‐ranging concept affected in a complex way by the person's physical health, psychological state, personal beliefs, social relationships and their relationship to salient features of their environment’^61^
Treatment‐related mortality	Any unexpected cause of death, which cannot be contributed to the disease itself, but can be explained by one of the applied MDS therapeutic interventions. This may include early death after induction or septicaemia due to prolonged cytopenia after drug therapy. The actual cause of death should be specified, if possible. Those deaths which can be explained by other conditions (e.g., stroke, heart attack, other malignancies, suicide, etc.) should be excluded
Overall survival	The length of time from the first MDS diagnosis until death, irrespective of the cause
Performance status	General definition: ‘The performance status describes the status of symptoms and functions with respect to ambulatory status and need for care’^38^
Safety	General definition: Safety can include assessment of the ‘adverse events, laboratory evaluations, vital signs’^60^, physical examinations, etc.
Haematological improvement	Definition according to the MDS IWG response criteria until the ongoing improvements become available

HMA, hypomethylating agents; IWG, International Working Group; MDS, myelodysplastic syndromes.

## Discussion

We developed the first comprehensive MDS‐specific COS, which recommends a minimum set of outcomes with the intention to increase comparability of study results and to reduce reporting bias and heterogeneity in outcome assessment. The newly developed MDS‐COS includes the following six core outcomes: HRQoL, treatment‐related mortality, OS, performance status, safety and haematological improvement. This set of outcomes should be reported in future clinical trials. However, primary and secondary outcomes of future studies still need to be defined separately, depending on the research question.

The findings from our study support and extend definitions of response criteria developed by the IWG taskforce.^4^, ^5^ The parameter ‘overall survival’ is identical both in the original^4^ and in the modified IWG criteria^5^ and in MDS‐COS. In contrast, the term ‘treatment‐related mortality’ from the MDS‐COS highlights the relevance of this parameter in the context of clinical trials and in HSCT, whereas in IWG^4^, ^5^ the more general term ‘event‐free survival’ is used, which includes events from any cause. The term ‘safety’ in MDS‐COS is clearly related to its application under the perspective of clinical studies. The parameter ‘haematological improvement’ in MDS‐COS summarises the different aspects of haematological responses, whereas many more details – such as changes in different cell lines in peripheral blood, reduction of bone marrow blasts and transfusion need, and cytogenetic response – are given in the IWG measures,^4^, ^5^ as well as in a recent summary of erythroid response.^28^ In general, the objectives of IWG response criteria are different from those of the MDS‐COS. IWG defined a broad range of clinician‐reported criteria, which are predominantly based on laboratory parameters and which are applied ‘for evaluating clinically significant responses in MDS’^5^ and to ‘define response criteria for altering natural history of MDS’ and were last updated in 2006.^5^ In contrast, the MDS‐COS represents a minimum reporting standard, which should be measured in each clinical trial to enhance comparability and enable quantitative evidence synthesis.

An important finding of our study is the inclusion of performance status and HRQoL in the MDS‐COS. Recent empirical data have shown a high prevalence of symptoms and functional limitations across all MDS disease‐risk group categories,^19^, ^20^ and also an independent association between self‐reported symptoms (i.e., fatigue) and survival outcomes, at least in higher‐risk disease patients.^29^ Indeed, the use of HRQoL, or other types of PROs, has been highly valued as a key outcome measure to better inform treatment decisions, not only in patients with MDS, but also in other cancer malignancies and to address safety aspects.^1^, ^30^, ^31^, ^32^, ^33^, ^34^ Significantly, the alleviation of disease‐related symptoms is of high importance and relevance for patients with MDS and, consequently, the integration of HRQoL as a clinical endpoint has been suggested by the IWG^4^ and by the recommendations from the European LeukemiaNet.^1^ Whereas measurement of HRQoL in specific domains has been suggested previously by the IWG^4^ and is receiving more and more attention, the implementation of HRQoL as response measurement may be challenging^35^ and more research in that area is needed.^33^ The COS suggested by the experts of EUMDS supports the relevance of integrating HRQoL into the list of outcomes and may further stimulate discussions on how to optimally implement HRQoL scoring systems in MDS studies. When measuring HRQoL, there is a trade‐off between using generic or disease‐specific instruments or even qualitative assessments. Generic instruments ‒ such as the EQ‐5D ‒ can be used for comparing HRQoL profiles across different diseases, as they are non‐specific for any medical condition. However, they may lack sensitivity in specific research settings, as they may not capture symptoms or functional aspects which are most relevant for a given patient population. On the other hand, HRQoL‐disease‐specific instruments can better assess key disease‐ and patient‐related symptoms as well as psychosocial aspects.^30^ In contrast, qualitative assessments can provide ‘an in‐depth understanding of patient experiences that may not otherwise be captured through the use of standardised questionnaires’.^36^ An advantage of the generic EQ‐5D instrument used in this study is that it also results in a single score, a so‐called utility, which can be directly implemented in health economic analyses, which evaluate quality‐adjusted life years gained by an intervention.

Performance status was ranked among the most relevant parameters by the clinical experts, thus achieving inclusion in MDS‐COS. So far, analyses on the relevance of performance status in patients with MDS are mainly restricted to its role as a prognostic factor for clinical outcome,^22^, ^37^ whereas its role in treatment response evaluation is relatively rare. Experts agreed on the following definition: ‘The performance status describes the status of symptoms and functions with respect to ambulatory status and need for care’.^38^ This description is in line with suggestions and data on the relevance of assessment of maintenance and improvement of functional capacities in the literature.^39^, ^40^ Thus, the evaluation of performance capacities should be extended beyond the assessment of performance status by the World Health Organisation, or Karnofsky‐Index, but should include scoring of functional activities and objective performance, based on a structured measurement as suggested by Hamaker *et al*.^41^ Likewise, the inclusion of tasks performed by the patient including a timed ‘up‐and‐go’ test or evaluation of gait speed have been suggested by panel recommendations from the American Society of Clinical Oncology^42^, and the European Organisation for Research and Treatment of Cancer and the International Society of Geriatric Oncology.^40^ Similarly, the US FDA has defined performance outcome measures as an essential part of clinical outcome assessment.^21^

The relevance of structured outcome‐reporting by the development of disease‐specific sets of parameters is in line with suggestions from the literature.^16^ Recommendations on consistent response criteria are available for several types of solid tumours and haematological malignancies.^16^, ^43^, ^44^, ^45^, ^46^, ^47^, ^48^, ^49^ Moreover, an agreed disease‐specific set of outcome parameters, known as COS, should be included in clinical studies. However, data on the definition and application of COS in malignant diseases are rare. We mainly identified COS in ovarian, prostate, head and neck cancer, as well as adult cancer treatment trials focusing on PROs.^16^ These studies focused on specific patient‐reported symptoms to be measured in cancer treatment trials.^50^, ^51^, ^52^, ^53^ Similarly to our COS development process, they followed a systematic approach to develop the core set of patient‐reported symptoms, including a systematic review and expert panel.

Overall, there is wide variation in the methods used for developing sets of standardised response criteria (e.g., semi‐structured group discussions, Delphi surveys).^47^ A strength of our development process was the systematic approach, which included several Delphi survey rounds with 75 experts from more than 15 different countries, ensuring a broad base of expertise.

Our study has several limitations. Our systematic literature search was limited to studies published in English with a pre‐specified time period in specific databases, which may have resulted in missing relevant studies.

Another limitation is that the goal of our study was to develop a COS for application in clinical studies. The methods for developing a COS for routine patient care or a registry study may differ from the currently used methods^54^, and different outcomes may have been included. Patients enrolled in clinical trials are usually younger, fitter, have fewer comorbidities and are closely monitored. Particularly in MDS patients, there may be significant differences between clinical trial populations and patients in routine clinical care and registries, as the median age at diagnosis of MDS patients is around 76 years.^55^ Additionally, specific outcomes may be more important for a specific patient subgroup. For example, HRQoL may be more important for lower‐risk MDS patients, while OS may be more important for higher‐risk MDS patients. Likewise, the timepoint when the outcomes are evaluated during the disease course may play an important role. The results of the first round of our Delphi survey show that the participants suggested some differences in the relevance of specific outcomes for specific situations. However, HRQoL, safety and haematological improvement did not show statistically significant differences related to the IPSS‐R risk. In addition, no statistically significant differences in the rankings were observed for HRQoL and OS regarding therapy and clinical setting, respectively. This similar relevance may indicate the broad applicability of the selected MDS core outcomes.

In general, as in all Delphi surveys, the results from our survey may be dependent on the composition of the panel.^56^ In our study, the survey group was limited to clinical experts in MDS, who may not represent the opinion of other relevant health service users (i.e., patients, regulators, and industry representatives) or policy decision makers (e.g., health technology assessment agencies, and reimbursement decision bodies). Additionally, the experts represent mainly the European setting. Healthcare systems from different regions may have different objectives and general conditions. Further, the inclusion of less experienced or less research‐oriented haematologists could add a perspective with more innovative approaches. As emphasised by the authors of the COMET handbook, the patients’ perspective should also be included to capture outcomes which are most relevant for patients.^54^ The currently developed MDS‐COS likely covers the most important patient‐relevant aspects with regard to HRQoL, patient‐reported aspects of safety, and performance status. However, the next step to complement our COS is an explicit inclusion of patients’ perspectives, by performing additional surveys and a validation in a patient population. Once these surveys are completed, those results may be integrated with the MDS‐COS. Since the relatively broad outcome of HRQoL is part of our MDS‐COS, we expect a more specific operationalisation from a patient survey.

Our project was the first step in establishing a MDS‐COS. For this first step, we included predominantly clinical experts who are mainly involved in the conduction of clinical trials, interpreting clinical trial results, applying knowledge derived from clinical trials and treating patients with MDS in daily routine care. In the future, the MDS‐COS should be continuously revised and updated in accordance with new therapeutic, pathological and molecular findings. A continuous discussion and update of the MDS‐COS with international MDS‐experts and stakeholders is planned and will widen its acceptability and acceptance. Moreover, adapting the general MDS‐COS specifically to different timepoints in the disease trajectory should be explored.

For the evaluation of the outcomes, the next important step is to establish recommendations regarding the tools for the measurement of each outcome ‒ for example, which score should be applied to measure performance status. In addition to the evaluation of outcomes, we plan to go a step further and will try to define the magnitude of a change in outcome which would be relevant and meaningful for patients. Benefits and harms, as well as the economic consequences of a new treatment strategy need to be carefully balanced to define a clinically relevant benefit for the patient.^57^ Additionally, for measurement, analysis and comparison of the different core outcomes, informative dropout and censoring needs to be considered. For example, longitudinal HRQoL comparisons may be biased when patients with a severe decrease in HRQoL miss having it evaluated, due to the worsened HRQoL.^58^ Therefore, it is important to minimise drop‐out, to collect information on informative dropout and censoring, and to consider the application of additional methods in order to correctly adjust for selection bias during follow‐up, such as causal inference generalised methods (g‐methods).^59^

We developed the first MDS‐COS by applying a comprehensive approach of systematic evidence synthesis, international survey process and consensus methods. This MDS‐COS includes the six outcomes: HRQoL, treatment‐related mortality, OS, performance status, safety, and haematological improvement. These outcomes are recommended to represent the minimum essential set, and should be reported as endpoints in future clinical MDS studies. The MDS‐COS aims to minimise the heterogeneity and inconsistency in outcome reporting, and increases the usability of study results in evidence synthesis and health technology assessments for clinicians and policy decision makers. In the future, the inclusion of further relevant stakeholders, continuous updating, and the evaluation of the acceptance of the MDS‐COS is recommended.

## Disclaimer

Results only reflect the author's view. The European Commission is not responsible for any use that may be made of the information it contains.

## Conflict of interest

Dr. Rochau reports other support from Erasmus Mundus Western Balkans (ERAWEB), a project funded by the European Commission, grants from the EU's Horizon 2020 research and innovation programme under grant agreement No. 634789, MDS‐RIGHT, within the Personalising Health and Care programme PHC‐2014‐634789, during the conduct of the study, and grants from Oncotyrol, outside the submitted work. Igor Stojkov reports other support from Erasmus Mundus Western Balkans (ERAWEB), a project funded by the European Commission, grants from the EU's Horizon 2020 research and innovation programme under grant agreement No. 634789, MDS‐RIGHT, within the Personalising Health and Care programme PHC‐2014‐634789, during the conduct of the study. Dr. Conrads‐Frank reports grants from the EU's Horizon 2020 research and innovation programme under grant agreement No. 634789, MDS‐RIGHT, within the Personalising Health and Care programme PHC‐2014‐634789, during the conduct of the study. Dr. Borba has nothing to disclose. Dr. Koinig reports grants from the EU's Horizon 2020 research and innovation programme and grants from the Austrian Science Fund during the conduct of the study. Dr. Arvandi reports grants from the EU’s Horizon 2020 research and innovation programme under grant agreement No. 634789, MDS‐RIGHT, within the Personalising Health and Care programme PHC‐2014‐634789, during the conduct of the study. Dr. van Marrewijk is funded from the EUMDS (educational grants from Novartis Oncology Europe, Amgen Limited, and Celgene International) and MDS‐RIGHT (grant from EU's Horizon 2020 program) project budgets. Dr. Garelius has nothing to disclose. Dr. Germing has nothing to disclose. Dr. Symeonidis reports institutional research support and other support from Abbvie, Amgen, Bristol‐Myers‐Squibb, Gilead, Janssen‐Cilag, Novartis, Roche, Sanofi/Genzyme and Takeda outside the submitted work. Dr. Sanz has nothing to disclose. Dr. Fenaux reports research support from Celgene, Astex, Jazz, Aprea, outside the submitted work. Dr. de Witte reports grants from the EU’s Horizon 2020 program, Novartis, Amgen and Celgene during the conduct of the study. Dr. Efficace reports personal fees from Bristol‐Myers Squibb, Amgen, Orsenix, Incyte and Takeda, and research funding (to his institution) from Amgen, outside the submitted work. Dr. Siebert reports other support from Erasmus Mundus Western Balkans (ERAWEB), a project funded by the European Commission, grants from the EU's Horizon 2020 research and innovation programme under grant agreement No. 634789, MDS‐RIGHT, within the Personalising Health and Care programme PHC‐2014‐634789, during the conduct of the study, and grants from Oncotyrol, outside the submitted work. Dr. Stauder reports other support from Celgene, Novartis and Teva outside the submitted work.

## Author contributions

Designed research: FE, IS, KK, RS, UR, US. Performed research: ACF, AS, CvM, FE, GS, HB, HG, IS, KK, MA, PF, RS, TdW, UG, UR, US. Collected data: ACF, HB, IS, UR, RS. Analysed and interpreted data: ACF, FE, HG, KK, MA, UG, RS, TdW, UR, US. Performed statistical analysis: IS, MA. Wrote the manuscript: IS, RS, UR, US. Reviewed the manuscript: ACF, AS, CvM, FE, GS, HB, HG, IS, KK, MA, PF, RS, TdW, UG, UR, US.

## Supporting information


**Table S1.** Study characteristics.
