# Supplementary material for: Development of a core outcome set for myelodysplastic syndromes – a Delphi study from the EUMDS Registry Group
Source: Br J Haematol. 2020 May 14;191(3):405–17. doi: 10.1111/bjh.16654 (PMC8221029; doi:10.1111/bjh.16654)
Supplement: Supplementary file 1 — Table S1. Study characteristics. [file BJH-191-405-s001.pdf]

Supplement British Journal of Haematology

Development of a Core Outcome Set for Myelodysplastic Syndromes – A Delphi Study from the EUMDS Registry Group

Rochau Ursula, MD[1], Stojkov Igor[1], MPharm, Conrads-Frank Annette[1], PhD, Borba Helena H[2], PhD, Koinig Karin A[3], PhD, Arvandi Marjan[1], PhD,van Marrewijk Corine[4], PhD, Garelius Hege[5], PhD, Germing Ulrich[6], MD, Symeonidis Argiris[7], PhD, Sanz Guillermo F[8,9], MD, Fenaux Pierre[10], MD, de Witte Theo[11], PhD, Efficace Fabio[12], PhD, Siebert Uwe, ScD[1,13,14], Stauder Reinhard[3], MD

[1] Institute of Public Health, Medical Decision Making and Health Technology Assessment, Department of Public Health, Health Services Research and Health Technology Assessment, UMIT – University for Health Sciences, Medical Informatics and Technology, Hall i.T., Austria

[2] Department of Pharmacy, Pharmaceutical Sciences Postgraduate Research Program, Federal University of Paraná, Curitiba, Brazil

[3] Department of Internal Medicine V (Hematology and Oncology), Innsbruck Medical University, Innsbruck, Austria

[4] Department of Hematology, Radboud university medical center, Nijmegen, Netherlands

[5] Department of Medicine, Section of Hematology and Coagulation, Sahlgrenska University Hospital, Göteborg, Sweden

[6] Department of Hematology, Oncology and Clinical Immunology, Universitätsklinik Düsseldorf, Düsseldorf, Germany

[7] Department of Internal Medicine, Div. Hematology, University of Patras Medical School, Patras, Greece

[8] Department of Hematology, Hospital Universitario y Politécnico La Fe, Valencia, Spain

[9] Centro de Investigación Biomédica en Red de Cáncer, CIBERONC, Instituto de Salud Carlos III, Madrid, Spain

[10] Service d'Hématologie, Hôpital Saint-Louis, Assistance Publique des Hôpitaux de Paris (AP-HP) and Université Paris 7, Paris, France

[11] Department of Tumor Immunology - Nijmegen Center for Molecular Life Sciences, Radboud university medical center, Nijmegen, Netherlands

[12] Health Outcomes Research Unit, Gruppo Italiano Malattie Ematologiche dell'Adulto (GIMEMA), Rome, Italy.

[13] Center for Health Decision Science, Department of Health Policy and Management, Harvard Chan School of Public Health, Boston, MA, USA

[14] Institute for Technology Assessment and Department of Radiology, Massachusetts General Hospital, Harvard Medical School, Boston, MA, USA

Address for correspondence:

Prof. Dr. Uwe Siebert, MPH, MSc

Center for Health Decision Science,

Department of Health Policy and Management,

Harvard T.H. Chan School of Public Health,

718 Huntington Avenue, Boston, MA, 02115-5924, USA

Tel: +43-50-8648-3930, Fax: +43-50-8648-673931

Email: usiebert@hsph.harvard.edu

Supplementary Table 1. Study characteristics

	Number of studies (%)
Study type	
CTP 0	4 (0.9)
CTP I	60 (14.1)
CTP I & II	58 (13.6)
CTP II	184 (43.3)
CTP II & III	6 (1.4)
CTP III	23 (5.4)
CTP IV	8 (1.9)
Observational study	9 (2.1)
Not stated	73 (17.2)
Examined intervention	
Medications	289 (68.0)
Transplantation	28 (6.6)
Drug & Transplantation	81 (19.1)
Transfusion	6 (1.4)
Procedures	6 (1.4)
Health/Molecular prognostic values	15 (3.5)
Sample size	
<30	98 (23.1)
30-100	222 (52.2)
>100	100 (23.5)
Not stated	5 (1.2)
Region	
North America	246 (57.9)
South America	2 (0.5)
Europe	66 (15.5)
Asia	73 (17.2)
Oceania	6 (1.4)
Intercontinental	32 (7.5)
Year	
2012	102 (24.0)
2013	105 (24.7)
2014	120 (28.2)
2015	96 (22.6)
2016	2 (0.5)
Enrolled patients	
Minimum one MDS patient among others	358 (84.2)
Solely MDS patients	67 (15.8)

CTP: Clinical Trial Phase.

DELPHI SURVEY

(First Round)

MDS-RIGHT - Providing the right care to the right patient with MyeloDysplastic Syndrome at the right time

Establishment of a new Core Outcome Set for MDS*

MDS-RIGHT project: Providing the right care to the right patient with MyeloDysplastic Syndrome at the right time*

The aim of our study is to identify and select the most relevant outcomes to be included in an MDS-specific core outcomes set (COS).

The COMET (Core Outcome Measures in Effectiveness Trials) initiative defined a COS as follows: “A core outcome set (COS) is an agreed minimum set of outcomes that should be measured and reported in all clinical trials, audits of practice or other forms of research for a specific condition” [1].

In the following section, we present 26 patient-relevant outcomes based on our previously performed MDS systematic literature search. For each outcome, please answer the following two categories:

1. Rank, on a scale from 1-9, the general importance of including the outcome in the COS for MDS patients:
   1-3 Low importance for decision-making
   4-6 Important, but not critical for decision-making
   7-9 Critical for decision-making

2. For each outcome, select the preferred/target MDS patient group/intervention and study type.

The survey will be used to identify and select important and critical outcomes. In further steps, these selected outcomes will be discussed and operationalized in detail in an expert panel.

Additional information:
https://mds-europe.eu/right
http://www.comet-initiative.org/

Technical support:
mdsonlinesurvey@gmail.com

*This work was supported by MDS-RIGHT: “Providing the right care to the right patient with MyeloDysplastic Syndrome at the right time”. The project has received funding from the European Union’s Horizon 2020 research and innovation programme under grant agreement No. 634789.

Establishment of a new Core Outcome Set for MDS

Welcome to the MDS Delphi survey.
Please complete the following questionnaire (participant's general information) and proceed to the next section.

*Required
Mark only one square.

General information from the participant:

1. Choose your work/practice country*.

☐ Austria ☐ France ☐ Italy ☐ Romania

☐ Croatia ☐ Germany ☐ Netherlands ☐ Serbia

☐ Czech Republic ☐ Greece ☐ Poland ☐ Spain

☐ Denmark ☐ Israel ☐ Portugal ☐ Sweden

☐ United Kingdom

2. Gender*

☐ Male ☐ Female

3. Age* ______

4. Specialty*

☐ Geriatric Medicine ☐ Hematology ☐ Oncology

☐ Hematology & Oncology ☐ Internal Medicine ☐ Others ______

5. **Work experience***

☐ <5 years ☐ 5-10 years ☐ >10 years

6. **Experience with MDS patients***

☐ <5 years ☐ 5-10 years ☐ >10 years

Please fill out both question categories for all outcomes. A general/non-MDS specific outcome definition has been provided for several of the outcomes.

Scale 1-9:
1-3 Low importance for decision-making
4-6 Important, but not critical for decision-making
7-9 Critical for decision-making
*Required

Overall survival

General definition: "The length of time from either the date of diagnosis or the start of treatment for a disease, that patients diagnosed with the disease are still alive" [2]. Death is considered irrespective of the cause.*

Mark only one number

Low importance for decision-making	1	2	3	4	5	6	7	8	9	Critical for decision-making

Patient risk group, intervention and study type where this outcome is most relevant

☐ Very low and low risk (IPSS-R 0-3)
☐ Intermediate risk (IPSS-R >3-4,5)
☐ High and very high risk (IPSS-R 4,5->6,5)
☐ Supportive therapy
☐ Disease-modifying therapy
☐ Hematopoietic stem cell transplantation
☐ Clinical study
☐ Registry
☐ Daily practice
☐ Other: ____________

Quality of life

Inclusion of the outcome into the general MDS COS:*

Mark only one number

Low importance for decision-making	1	2	3	4	5	6	7	8	9	Critical for decision-making

Patient risk group, intervention and study type where this outcome is most relevant

☐ Very low and low risk (IPSS-R 0-3)
☐ Intermediate risk (IPSS-R >3-4,5)
☐ High and very high risk (IPSS-R 4,5->6,5)
☐ Supportive therapy
☐ Disease-modifying therapy
☐ Hematopoietic stem cell transplantation
☐ Clinical study
☐ Registry

☐ Daily practice
☐ Other: ______________________

Duration of hospitalization

Inclusion of the outcome into the general MDS COS:*

Mark only one number

Low importance for decision-making	1	2	3	4	5	6	7	8	9	Critical for decision-making

Patient risk group, intervention and study type where this outcome is most relevant

☐ Very low and low risk (IPSS-R 0-3)
☐ Intermediate risk (IPSS-R >3-4,5)
☐ High and very high risk (IPSS-R 4,5->6,5)
☐ Supportive therapy
☐ Disease-modifying therapy
☐ Hematopoietic stem cell transplantation
☐ Clinical study
☐ Registry
☐ Daily practice
☐ Other: ______________________

Cytogenetic response

Inclusion of the outcome into the general MDS COS:*

Mark only one number

Low importance for decision-making	1	2	3	4	5	6	7	8	9	Critical for decision-making

Patient risk group, intervention and study type where this outcome is most relevant

☐ Very low and low risk (IPSS-R 0-3)
☐ Intermediate risk (IPSS-R >3-4,5)
☐ High and very high risk (IPSS-R 4,5->6,5)
☐ Supportive therapy
☐ Disease-modifying therapy
☐ Hematopoietic stem cell transplantation
☐ Clinical study
☐ Registry
☐ Daily practice
☐ Other: ______________________

Hematological improvement

General definition: Haematological improvements of cytopenias.*

Mark only one number

Low importance for decision-making	1	2	3	4	5	6	7	8	9	Critical for decision-making

Patient risk group, intervention and study type where this outcome is most relevant

☐ Very low and low risk (IPSS-R 0-3)
☐ Intermediate risk (IPSS-R >3-4,5)
☐ High and very high risk (IPSS-R 4,5->6,5)
☐ Supportive therapy
☐ Disease-modifying therapy
☐ Hematopoietic stem cell transplantation
☐ Clinical study
☐ Registry
☐ Daily practice
☐ Other: ____________

Response/Remission

General definition: Remission in terms of complete remission, partial remission and/or marrow remission etc.*

Mark only one number

Low importance for decision-making	1	2	3	4	5	6	7	8	9	Critical for decision-making

Patient risk group, intervention and study type where this outcome is most relevant

☐ Very low and low risk (IPSS-R 0-3)
☐ Intermediate risk (IPSS-R >3-4,5)
☐ High and very high risk (IPSS-R 4,5->6,5)
☐ Supportive therapy
☐ Disease-modifying therapy
☐ Hematopoietic stem cell transplantation
☐ Clinical study
☐ Registry
☐ Daily practice
☐ Other: ____________

Time to response

Inclusion of the outcome into the general MDS COS:*

Mark only one number

Low importance for decision-making	1	2	3	4	5	6	7	8	9	Critical for decision-making

Patient risk group, intervention and study type where this outcome is most relevant

☐ Very low and low risk (IPSS-R 0-3)
☐ Intermediate risk (IPSS-R >3-4,5)
☐ High and very high risk (IPSS-R 4,5->6,5)
☐ Supportive therapy
☐ Disease-modifying therapy
☐ Hematopoietic stem cell transplantation
☐ Clinical study
☐ Registry
☐ Daily practice
☐ Other: ________________

Overall response

General definition: Summary of complete, partial, marrow and/or hematological responses.*

Mark only one number

Low importance for decision-making	1	2	3	4	5	6	7	8	9	Critical for decision-making

Patient risk group, intervention and study type where this outcome is most relevant

☐ Very low and low risk (IPSS-R 0-3)
☐ Intermediate risk (IPSS-R >3-4,5)
☐ High and very high risk (IPSS-R 4,5->6,5)
☐ Supportive therapy
☐ Disease-modifying therapy
☐ Hematopoietic stem cell transplantation
☐ Clinical study
☐ Registry
☐ Daily practice
☐ Other: ________________

Safety

General definition: Safety can include: adverse events, laboratory evaluations, vital signs, physical examinations etc. [3]*

Mark only one number

Low importance for decision-making	1	2	3	4	5	6	7	8	9	Critical for decision-making

Patient risk group, intervention and study type where this outcome is most relevant

☐ Very low and low risk (IPSS-R 0-3)
☐ Intermediate risk (IPSS-R >3-4,5)
☐ High and very high risk (IPSS-R 4,5->6,5)
☐ Supportive therapy

☐ Disease-modifying therapy
☐ Hematopoietic stem cell transplantation
☐ Clinical study
☐ Registry
☐ Daily practice
☐ Other: ______________

Adverse event

General definition: Any occurrence or worsening of an undesirable or unintended sign, symptom (including an abnormal laboratory finding), or disease temporally associated with the use of a medicinal product/procedure, whether or not related to the medicinal product/procedure [4].*

Mark only one number

Low importance for decision-making	1	2	3	4	5	6	7	8	9	Critical for decision-making

Patient risk group, intervention and study type where this outcome is most relevant

☐ Very low and low risk (IPSS-R 0-3)
☐ Intermediate risk (IPSS-R >3-4,5)
☐ High and very high risk (IPSS-R 4,5->6,5)
☐ Supportive therapy
☐ Disease-modifying therapy
☐ Hematopoietic stem cell transplantation
☐ Clinical study
☐ Registry
☐ Daily practice
☐ Other: ______________

Infectious event

Inclusion of the outcome into the general MDS COS:*

Mark only one number

Low importance for decision-making	1	2	3	4	5	6	7	8	9	Critical for decision-making

Patient risk group, intervention and study type where this outcome is most relevant

☐ Very low and low risk (IPSS-R 0-3)
☐ Intermediate risk (IPSS-R >3-4,5)
☐ High and very high risk (IPSS-R 4,5->6,5)
☐ Supportive therapy
☐ Disease-modifying therapy
☐ Hematopoietic stem cell transplantation
☐ Clinical study
☐ Registry
☐ Daily practice
☐ Other: ______________

Iron overload

General definition: Iron overload indicates excess of iron in the body from any cause. This can be defined by evaluation of serum ferritin and transferrin saturation or if available by magnetic resonance imaging [5].*

Mark only one number

Low importance for decision-making	1	2	3	4	5	6	7	8	9	Critical for decision-making

Patient risk group, intervention and study type where this outcome is most relevant

☐ Very low and low risk (IPSS-R 0-3)
☐ Intermediate risk (IPSS-R >3-4,5)
☐ High and very high risk (IPSS-R 4,5->6,5)
☐ Supportive therapy
☐ Disease-modifying therapy
☐ Hematopoietic stem cell transplantation
☐ Clinical study
☐ Registry
☐ Daily practice
☐ Other: ________________

Secondary morbidity

General definition: The occurrence of morbidities after the time of diagnosing and/or treating the patient with MDS*

Mark only one number

Low importance for decision-making	1	2	3	4	5	6	7	8	9	Critical for decision-making

Patient risk group, intervention and study type where this outcome is most relevant

☐ Very low and low risk (IPSS-R 0-3)
☐ Intermediate risk (IPSS-R >3-4,5)
☐ High and very high risk (IPSS-R 4,5->6,5)
☐ Supportive therapy
☐ Disease-modifying therapy
☐ Hematopoietic stem cell transplantation
☐ Clinical study
☐ Registry
☐ Daily practice
☐ Other: ________________

Need for supportive therapy

General definition: The necessity of implementing supportive therapy among MDS patients (e.g., transfusions of erythrocytes and/or thrombocytes, interventional G-CSF, antibiotic therapy etc.) [6].*

Mark only one number

Low importance for decision-making	1	2	3	4	5	6	7	8	9	Critical for decision-making

Patient risk group, intervention and study type where this outcome is most relevant

☐ Very low and low risk (IPSS-R 0-3)
☐ Intermediate risk (IPSS-R >3-4,5)
☐ High and very high risk (IPSS-R 4,5->6,5)
☐ Supportive therapy
☐ Disease-modifying therapy
☐ Hematopoietic stem cell transplantation
☐ Clinical study
☐ Registry
☐ Daily practice
☐ Other: ____________________

Treatment-related mortality

Inclusion of the outcome into the general MDS COS:*

Mark only one number

Low importance for decision-making	1	2	3	4	5	6	7	8	9	Critical for decision-making

Patient risk group, intervention and study type where this outcome is most relevant

☐ Very low and low risk (IPSS-R 0-3)
☐ Intermediate risk (IPSS-R >3-4,5)
☐ High and very high risk (IPSS-R 4,5->6,5)
☐ Supportive therapy
☐ Disease-modifying therapy
☐ Hematopoietic stem cell transplantation
☐ Clinical study
☐ Registry
☐ Daily practice
☐ Other: ____________________

Acute/Chronic graft-versus-host disease

Inclusion of the outcome into the general MDS COS:*

Mark only one number

Low importance for decision-making	1	2	3	4	5	6	7	8	9	Critical for decision-making

Patient risk group, intervention and study type where this outcome is most relevant

☐ Very low and low risk (IPSS-R 0-3)
☐ Intermediate risk (IPSS-R >3-4,5)
☐ High and very high risk (IPSS-R 4,5->6,5)
☐ Supportive therapy
☐ Disease-modifying therapy
☐ Hematopoietic stem cell transplantation
☐ Clinical study
☐ Registry
☐ Daily practice
☐ Other: ____________

Event-free survival

General definition: The length of time from either the date of diagnosis or the start of treatment for a disease until experiencing a particular group of predefined events (e.g., fracture, infection, laboratory test abnormality, particular kind of progression, death from any cause) [7].*

Mark only one number

Low importance for decision-making	1	2	3	4	5	6	7	8	9	Critical for decision-making

Patient risk group, intervention and study type where this outcome is most relevant

☐ Very low and low risk (IPSS-R 0-3)
☐ Intermediate risk (IPSS-R >3-4,5)
☐ High and very high risk (IPSS-R 4,5->6,5)
☐ Supportive therapy
☐ Disease-modifying therapy
☐ Hematopoietic stem cell transplantation
☐ Clinical study
☐ Registry
☐ Daily practice
☐ Other: ____________

Failure-free survival

General definition: The length of time from either the date of diagnosis or the start of treatment for a disease until disease progression, recurrence during predefined line treatment, absence of additional line of therapy, death during treatment, death from any cause etc. [8].*

Mark only one number

Low importance for decision-making	1	2	3	4	5	6	7	8	9	Critical for decision-making

Patient risk group, intervention and study type where this outcome is most relevant

☐ Very low and low risk (IPSS-R 0-3)
☐ Intermediate risk (IPSS-R >3-4,5)
☐ High and very high risk (IPSS-R 4,5->6,5)
☐ Supportive therapy
☐ Disease-modifying therapy
☐ Hematopoietic stem cell transplantation
☐ Clinical study
☐ Registry
☐ Daily practice
☐ Other: ____________

Disease-free survival

General definition: The length of time from either the date of diagnosis or the start of treatment for a disease until recurrence of the disease, diagnosing other diseases (related/not related to the primary disease of interest) or death from any cause [9].*

Mark only one number

Low importance for decision-making	1	2	3	4	5	6	7	8	9	Critical for decision-making

Patient risk group, intervention and study type where this outcome is most relevant

☐ Very low and low risk (IPSS-R 0-3)
☐ Intermediate risk (IPSS-R >3-4,5)
☐ High and very high risk (IPSS-R 4,5->6,5)
☐ Supportive therapy
☐ Disease-modifying therapy
☐ Hematopoietic stem cell transplantation
☐ Clinical study
☐ Registry
☐ Daily practice
☐ Other: ____________

Relapse-free survival

General definition: The length of time from either the date of diagnosis or the start of treatment for a disease until disease recurrence or death from any cause [10].*

Mark only one number

Low importance for decision-making	1	2	3	4	5	6	7	8	9	Critical for decision-making

Patient risk group, intervention and study type where this outcome is most relevant

☐ Very low and low risk (IPSS-R 0-3)
☐ Intermediate risk (IPSS-R >3-4,5)

☐ High and very high risk (IPSS-R 4,5->6,5)
☐ Supportive therapy
☐ Disease-modifying therapy
☐ Hematopoietic stem cell transplantation
☐ Clinical study
☐ Registry
☐ Daily practice
☐ Other: ____________

Progression-free survival

General definition: The length of time from either the date of diagnosis or the start of treatment for a disease until disease progression or death, irrespective of the cause [11].*

Mark only one number

Low importance for decision-making	1	2	3	4	5	6	7	8	9	Critical for decision-making

Patient risk group, intervention and study type where this outcome is most relevant

☐ Very low and low risk (IPSS-R 0-3)
☐ Intermediate risk (IPSS-R >3-4,5)
☐ High and very high risk (IPSS-R 4,5->6,5)
☐ Supportive therapy
☐ Disease-modifying therapy
☐ Hematopoietic stem cell transplantation
☐ Clinical study
☐ Registry
☐ Daily practice
☐ Other: ____________

Time to progression

General definition: The length of time from either the date of diagnosis or the start of treatment for a disease until disease progression or death due to disease progression [11].*

Mark only one number

Low importance for decision-making	1	2	3	4	5	6	7	8	9	Critical for decision-making

Patient risk group, intervention and study type where this outcome is most relevant

☐ Very low and low risk (IPSS-R 0-3)
☐ Intermediate risk (IPSS-R >3-4,5)
☐ High and very high risk (IPSS-R 4,5->6,5)
☐ Supportive therapy
☐ Disease-modifying therapy
☐ Hematopoietic stem cell transplantation

☐ Clinical study
☐ Registry
☐ Daily practice
☐ Other: ______________

Performance status

General definition: The performance status describes the status of symptoms and functions with respect to ambulatory status and need for care [12].*

Mark only one number

Low importance for decision-making	1	2	3	4	5	6	7	8	9	Critical for decision-making

Patient risk group, intervention and study type where this outcome is most relevant

☐ Very low and low risk (IPSS-R 0-3)
☐ Intermediate risk (IPSS-R >3-4,5)
☐ High and very high risk (IPSS-R 4,5->6,5)
☐ Supportive therapy
☐ Disease-modifying therapy
☐ Hematopoietic stem cell transplantation
☐ Clinical study
☐ Registry
☐ Daily practice
☐ Other: ______________

Functional activities

General definition: Functional activities include activities of daily living (e.g., bathing, dressing, eating) and instrumental activities of daily living which defines functions which are essential to live autonomously (e.g., making household, preparing meals, making phone calls or other communication, taking prescribed medications etc.) [13].*

Mark only one number

Low importance for decision-making	1	2	3	4	5	6	7	8	9	Critical for decision-making

Patient risk group, intervention and study type where this outcome is most relevant

☐ Very low and low risk (IPSS-R 0-3)
☐ Intermediate risk (IPSS-R >3-4,5)
☐ High and very high risk (IPSS-R 4,5->6,5)
☐ Supportive therapy
☐ Disease-modifying therapy
☐ Hematopoietic stem cell transplantation
☐ Clinical study
☐ Registry

☐ Daily practice
☐ Other: ______________________

Number of transfusions per patient

Inclusion of the outcome into the general MDS COS:*

Mark only one number

Low importance for decision-making	1	2	3	4	5	6	7	8	9	Critical for decision-making

Patient risk group, intervention and study type where this outcome is most relevant

☐ Very low and low risk (IPSS-R 0-3)
☐ Intermediate risk (IPSS-R >3-4,5)
☐ High and very high risk (IPSS-R 4,5->6,5)
☐ Supportive therapy
☐ Disease-modifying therapy
☐ Hematopoietic stem cell transplantation
☐ Clinical study
☐ Registry
☐ Daily practice
☐ Other: ______________________

Need for hematopoietic stem cell transplantation

Inclusion of the outcome into the general MDS COS:*

Mark only one number

Low importance for decision-making	1	2	3	4	5	6	7	8	9	Critical for decision-making

Patient risk group, intervention and study type where this outcome is most relevant

☐ Very low and low risk (IPSS-R 0-3)
☐ Intermediate risk (IPSS-R >3-4,5)
☐ High and very high risk (IPSS-R 4,5->6,5)
☐ Supportive therapy
☐ Disease-modifying therapy
☐ Hematopoietic stem cell transplantation
☐ Clinical study
☐ Registry
☐ Daily practice
☐ Other: ______________________

Additional outcomes?

Comments/suggestions?

Thank you for taking the time to fill out this survey!

Filling out and handing in the survey indicates that:

• you have read and understood the above mentioned project description and instructions
• you voluntarily agree to participate
• you are at least 18 years of age
• you fill and submit the survey only once
• you agree that the information you provided is stored, analyzed and may be used for publication

If you do not wish to participate in the survey, feel free to decline your participation.

References

1. Prinsen, C.A.C., et al., Core Outcome Measures in Effectiveness Trials (COMET) initiative: protocol for an international Delphi study to achieve consensus on how to select outcome measurement instruments for outcomes included in a ‘core outcome set’. Trials, 2014. 15(1): p. 1-7.
2. NCI Dictionary of Cancer Terms. National Cancer Institute (NCI). U.S. Department of Health and Human Services. National Institutes of Health (NIH).
3. Gad, Shayne Cox. Drug Safety Evaluation. Pharmaceutical Development Series (2nd ed.). 2009;
4. Norman M. Goldfarb, Adverse Event Terminology, 8 J. Clinical Research Best Practices 1. 2012;
5. Hider, Robert C.; Kong, Xiaole (2013). "Chapter 8. Iron: Effect of Overload and Deficiency". In Astrid Sigel, Helmut Sigel and Roland K. O. Sigel. Interrelations between Essential Metal Ions and Human Diseases. Metal Ions in Life Sciences 13. Springer. pp. 229–294.
6. Scott, B.L. and E.L.I. Estey, Management of Myelodysplastic Syndromes: 2008 Update. Oncology (Williston Park, N.Y.), 2008. 22(12): p. 1344-1368
7. Indranil Mallick, M. Leukemia&Lymphoma: Event-Free Survival (EFS). 2016;
8. Palmer, J., et al., Failure-free survival in a prospective cohort of patients with chronic graft-versus-host disease. Haematologica, 2015. 100(5): p. 690-695.
9. U.S. Department of Health and Human Services Food and Drug Administration Center for Drug Evaluation and Research (CDER), Center for Biologics Evaluation and Research (CBER). Guidance for industry: clinical trial endpoints for the approval of cancer drugs and biologics. 2007;
10. Hudis CA, Barlow WE, Costantino JP, et al. Proposal for standardized definitions for efficacy end points in adjuvant breast cancer trials: the STEEP system. J Clin Oncol 2007;25(15):2127-2132
11. Kyle, R.A. and S.V. Rajkumar, Criteria for diagnosis, staging, risk stratification and response assessment of multiple myeloma. Leukemia : official journal of the Leukemia Society of America, Leukemia Research Fund, U.K, 2009. 23(1): p.3-9.
12. Sørensen, J.B., et al., Performance status assessment in cancer patients. An inter-observer variability study. British Journal of Cancer, 1993. 67(4): p. 773-775.
13. Activities of Daily Living Evaluation." Encyclopedia of Nursing & Allied Health. ed. Kristine Krapp. Gale Group, Inc., 2002. eNotes.com. 2006

DELPHI SURVEY

(Second Round)

MDS-RIGHT - Providing the right care to the right patient with MyeloDysplastic Syndrome at the right time

Establishment of a new Core Outcome Set for MDS* (Second round)

MDS-RIGHT project: Providing the right care to the right patient with MyeloDysplastic Syndrome at the right time*

The aim of our study is to identify and select the most relevant outcomes to be included in an MDS-specific core outcomes set (COS).

The COMET (Core Outcome Measures in Effectiveness Trials) initiative defined a COS as follows: “A core outcome set (COS) is an agreed minimum set of outcomes that should be measured and reported in all clinical trials, audits of practice or other forms of research for a specific condition” [1].

In this second round, we present 26 patient- and clinically-relevant outcomes based on our previously performed MDS systematic literature search and one additional outcome, which has been included as a suggestion of at least two participants ("Secondary malignancy"). For each outcome, please answer the following question:

1. Rank, on a scale from 1-9, the general importance of including the outcome in the COS for MDS patients:
   1-3 Low importance for decision-making
   4-6 Important, but not critical for decision-making
   7-9 Critical for decision-making

This second round should enable the participants to reconsider their opinion based on the group's results from the first round. The results of the survey will be used to identify and select important and critical outcomes. In further steps, these selected outcomes will be discussed and operationalized in detail in an expert panel.

Additional information:
https://mds-europe.eu/right
http://www.comet-initiative.org/

Technical support:
mdsonlinesurvey@gmail.com

*This work was supported by MDS-RIGHT: “Providing the right care to the right patient with MyeloDysplastic Syndrome at the right time”. The project has received funding from the European Union’s Horizon 2020 research and innovation programme under grant agreement No. 634789.

Establishment of a new Core Outcome Set for MDS

(Second round)

Welcome to the MDS Delphi survey.
Please complete the following questionnaire (participant's general information) and proceed to the next section.

*Required
Mark only one square.

General information from the participant:

1. Choose your work/practice country*.

☐ Austria ☐ France ☐ Italy ☐ Romania

☐ Croatia ☐ Germany ☐ Netherlands ☐ Serbia

☐ Czech Republic ☐ Greece ☐ Poland ☐ Spain

☐ Denmark ☐ Israel ☐ Portugal ☐ Sweden

☐ United Kingdom

2. Gender*

☐ Male ☐ Female

3. Age* ________

4. Specialty*

☐ Geriatric Medicine ☐ Hematology ☐ Oncology

☐ Hematology & Oncology ☐ Internal Medicine ☐ Others ________

5. **Work experience***

☐ <5 years ☐ 5-10 years ☐ >10 years

6. **Experience with MDS patients***

☐ <5 years ☐ 5-10 years ☐ >10 years

7. Did you participate in the previous, first round of the MDS-survey?*

☐ Yes ☐ No

Figure 1 Results from the first round of the MDS-COS survey

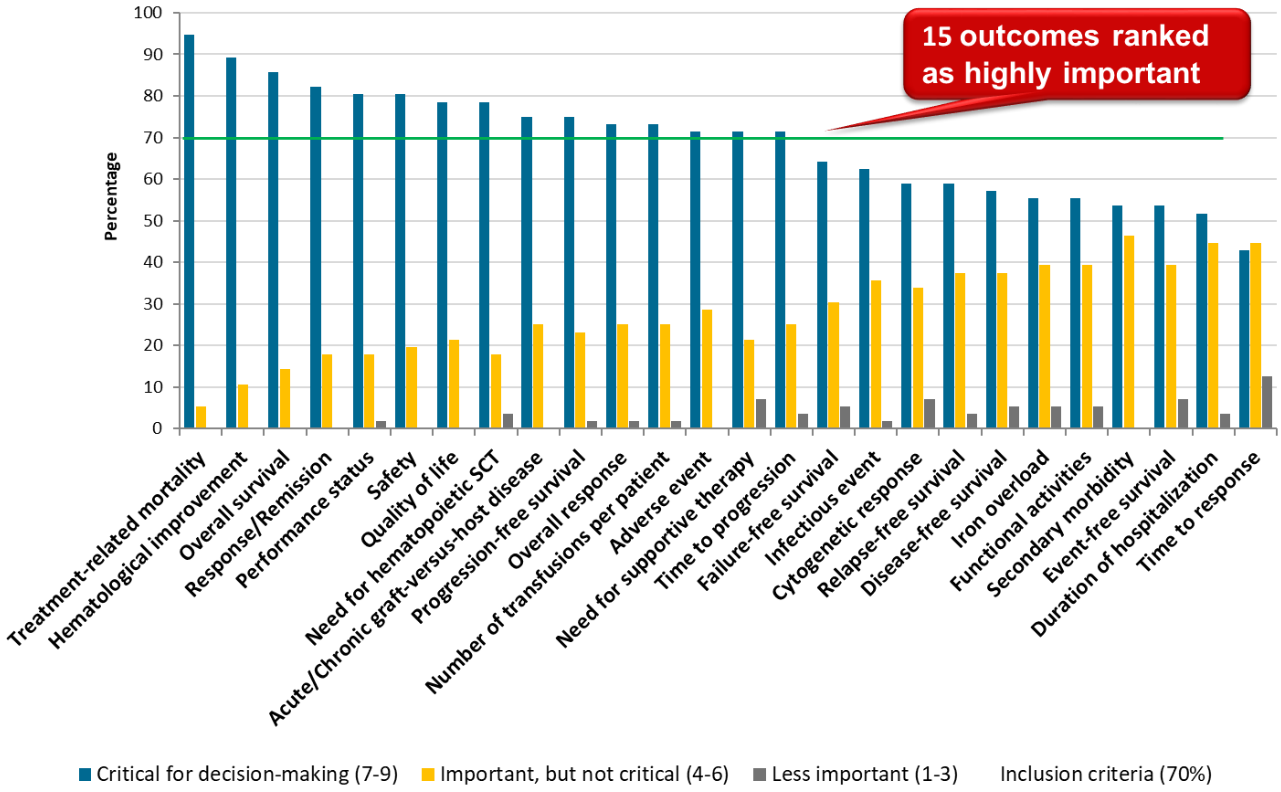

Please fill out the questions for all outcomes. A general/non-MDS specific outcome definition has been provided for several of the outcomes.

Scale 1-9:
1-3 Low importance for decision-making
4-6 Important, but not critical for decision-making
7-9 Critical for decision-making
*Required

Overall survival

General definition: "The length of time from either the date of diagnosis or the start of treatment for a disease, that patients diagnosed with the disease are still alive" [2]. Death is considered irrespective of the cause.*

Mark only one number

Low importance for decision-making	1	2	3	4	5	6	7	8	9	Critical for decision-making

Quality of life

Inclusion of the outcome into the general MDS COS:*

Mark only one number

Low importance for decision-making	1	2	3	4	5	6	7	8	9	Critical for decision-making

Duration of hospitalization

Inclusion of the outcome into the general MDS COS:*

Mark only one number

Low importance for decision-making	1	2	3	4	5	6	7	8	9	Critical for decision-making

Cytogenetic response

Inclusion of the outcome into the general MDS COS:*

Mark only one number

Low importance for decision-making	1	2	3	4	5	6	7	8	9	Critical for decision-making

Hematological improvement

General definition: Haematological improvements of cytopenias.*

Mark only one number

Low importance for decision-making	1	2	3	4	5	6	7	8	9	Critical for decision-making

Response/Remission

General definition: Remission in terms of complete remission, partial remission and/or marrow remission etc.*

Mark only one number

Low importance for decision-making	1	2	3	4	5	6	7	8	9	Critical for decision-making

Time to response

Inclusion of the outcome into the general MDS COS:*

Mark only one number

Low importance for decision-making	1	2	3	4	5	6	7	8	9	Critical for decision-making

Overall response

General definition: Summary of complete, partial, marrow and/or hematological responses.*

Mark only one number

Low importance for decision-making	1	2	3	4	5	6	7	8	9	Critical for decision-making

Safety

General definition: Safety can include: adverse events, laboratory evaluations, vital signs, physical examinations etc. [3].*

Mark only one number

Low importance for decision-making	1	2	3	4	5	6	7	8	9	Critical for decision-making

Adverse event

General definition: Any occurrence or worsening of an undesirable or unintended sign, symptom (including an abnormal laboratory finding), or disease temporally associated with the use of a medicinal product/procedure, whether or not related to the medicinal product/procedure [4].*

Mark only one number

Low importance for decision-making	1	2	3	4	5	6	7	8	9	Critical for decision-making

Infectious event

Inclusion of the outcome into the general MDS COS:*

Mark only one number

Low importance for decision-making	1	2	3	4	5	6	7	8	9	Critical for decision-making

Iron overload

General definition: Iron overload indicates excess of iron in the body from any cause. This can be defined by evaluation of serum ferritin and transferrin saturation or if available by magnetic resonance imaging [5].*

Mark only one number

Low importance for decision-making	1	2	3	4	5	6	7	8	9	Critical for decision-making

Secondary morbidity

General definition: The occurrence of morbidities after the time of diagnosing and/or treating the patient with MDS*

Mark only one number

Low importance for decision-making	1	2	3	4	5	6	7	8	9	Critical for decision-making

Need for supportive therapy

General definition: The necessity of implementing supportive therapy among MDS patients (e.g., transfusions of erythrocytes and/or thrombocytes, interventional G-CSF, antibiotic therapy etc.) [6].*

Mark only one number

Low importance for decision-making	1	2	3	4	5	6	7	8	9	Critical for decision-making

Treatment-related mortality

Inclusion of the outcome into the general MDS COS:*

Mark only one number

Low importance for decision-making	1	2	3	4	5	6	7	8	9	Critical for decision-making

Acute/Chronic graft-versus-host disease

Inclusion of the outcome into the general MDS COS:*

Mark only one number

Low importance for decision-making	1	2	3	4	5	6	7	8	9	Critical for decision-making

Event-free survival

General definition: The length of time from either the date of diagnosis or the start of treatment for a disease until experiencing a particular group of predefined events (e.g., fracture, infection, laboratory test abnormality, particular kind of progression, death from any cause) [7].*

Mark only one number

Low importance for decision-making	1	2	3	4	5	6	7	8	9	Critical for decision-making

Failure-free survival

General definition: The length of time from either the date of diagnosis or the start of treatment for a disease until disease progression, recurrence during predefined line treatment, absence of additional line of therapy, death during treatment, death from any cause etc. [8].*

Mark only one number

Low importance for decision-making	1	2	3	4	5	6	7	8	9	Critical for decision-making

Disease-free survival

General definition: The length of time from either the date of diagnosis or the start of treatment for a disease until recurrence of the disease, diagnosing other diseases (related/not related to the primary disease of interest) or death from any cause [9].*

Mark only one number

Low importance for decision-making	1	2	3	4	5	6	7	8	9	Critical for decision-making

Relapse-free survival

General definition: The length of time from either the date of diagnosis or the start of treatment for a disease until disease recurrence or death from any cause [10].*

Mark only one number

Low importance for decision-making	1	2	3	4	5	6	7	8	9	Critical for decision-making

Progression-free survival

General definition: The length of time from either the date of diagnosis or the start of treatment for a disease until disease progression or death, irrespective of the cause [11].*

Mark only one number

Low importance for decision-making	1	2	3	4	5	6	7	8	9	Critical for decision-making

Time to progression

General definition: The length of time from either the date of diagnosis or the start of treatment for a disease until disease progression or death due to disease progression [11].*

Mark only one number

Low importance for decision-making	1	2	3	4	5	6	7	8	9	Critical for decision-making

Performance status

General definition: The performance status describes the status of symptoms and functions with respect to ambulatory status and need for care [12].*

Mark only one number

Low importance for decision-making	1	2	3	4	5	6	7	8	9	Critical for decision-making

Functional activities

General definition: Functional activities include activities of daily living (e.g., bathing, dressing, eating) and instrumental activities of daily living which defines functions which are essential to live autonomously (e.g., making household, preparing meals, making phone calls or other communication, taking prescribed medications etc.) [13].*

Mark only one number

Low importance for decision-making	1	2	3	4	5	6	7	8	9	Critical for decision-making

Number of transfusions per patient

Inclusion of the outcome into the general MDS COS:*

Mark only one number

Low importance for decision-making	1	2	3	4	5	6	7	8	9	Critical for decision-making

Need for hematopoietic stem cell transplantation

Inclusion of the outcome into the general MDS COS:*

Mark only one number

Low importance for decision-making	1	2	3	4	5	6	7	8	9	Critical for decision-making

Secondary malignancy

General definition: Secondary malignancy could be defined as a new cancer that has occurred as a result of previous treatment with radiation or chemotherapy [14].*

Mark only one number

Low importance for decision-making	1	2	3	4	5	6	7	8	9	Critical for decision-making

Additional outcomes?

Comments/suggestions?

Thank you for taking the time to fill out this survey!

Filling out and handing in the survey indicates that:

- you have read and understood the above mentioned project description and instructions
- you voluntarily agree to participate
- you are at least 18 years of age
- you fill and submit the survey only once
- you agree that the information you provided is stored, analyzed and may be used for publication

If you do not wish to participate in the survey, feel free to decline your participation.

References

1. Prinsen, C.A.C., et al., Core Outcome Measures in Effectiveness Trials (COMET) initiative: protocol for an international Delphi study to achieve consensus on how to select outcome measurement instruments for outcomes included in a ‘core outcome set’. Trials, 2014. 15(1): p. 1-7.
2. NCI Dictionary of Cancer Terms. National Cancer Institute (NCI). U.S. Department of Health and Human Services. National Institutes of Health (NIH).
3. Gad, Shayne Cox. Drug Safety Evaluation. Pharmaceutical Development Series (2nd ed.). 2009;
4. Norman M. Goldfarb, Adverse Event Terminology, 8 J. Clinical Research Best Practices 1. 2012;
5. Hider, Robert C.; Kong, Xiaole (2013). "Chapter 8. Iron: Effect of Overload and Deficiency". In Astrid Sigel, Helmut Sigel and Roland K. O. Sigel. Interrelations between Essential Metal Ions and Human Diseases. Metal Ions in Life Sciences 13. Springer. pp. 229–294.
6. Scott, B.L. and E.L.I. Estey, Management of Myelodysplastic Syndromes: 2008 Update. Oncology (Williston Park, N.Y.), 2008. 22(12): p. 1344-1368
7. Indranil Mallick, M. Leukemia&Lymphoma: Event-Free Survival (EFS). 2016;
8. Palmer, J., et al., Failure-free survival in a prospective cohort of patients with chronic graft-versus-host disease. Haematologica, 2015. 100(5): p. 690-695.
9. U.S. Department of Health and Human Services Food and Drug Administration Center for Drug Evaluation and Research (CDER), Center for Biologics Evaluation and Research (CBER). Guidance for industry: clinical trial endpoints for the approval of cancer drugs and biologics. 2007;
10. Hudis CA, Barlow WE, Costantino JP, et al. Proposal for standardized definitions for efficacy end points in adjuvant breast cancer trials: the STEEP system. J Clin Oncol 2007;25(15):2127-2132
11. Kyle, R.A. and S.V. Rajkumar, Criteria for diagnosis, staging, risk stratification and response assessment of multiple myeloma. Leukemia : official journal of the Leukemia Society of America, Leukemia Research Fund, U.K, 2009. 23(1): p.3-9.
12. Sørensen, J.B., et al., Performance status assessment in cancer patients. An inter-observer variability study. British Journal of Cancer, 1993. 67(4): p. 773-775.
13. Activities of Daily Living Evaluation." Encyclopedia of Nursing & Allied Health. ed. Kristine Krapp. Gale Group, Inc., 2002. eNotes.com. 2006
14. Bagri PK, Singh D, Singhal MK, et al. Double Primary Malignancies: A Clinical & Pathological Analysis Report from a Regional Cancer Institute in India. Iranian Journal of Cancer Prevention. 2014;7(2):66-72.
